# Application of amodal segmentation for shape reconstruction and occlusion recovery in occluded tomatoes

**DOI:** 10.3389/fpls.2024.1376138

**Published:** 2024-06-13

**Authors:** Jing Yang, Hanbing Deng, Yufeng Zhang, Yuncheng Zhou, Teng Miao

**Affiliations:** ^1^ College of Information and Electrical Engineering, Shenyang Agricultural University, Shenyang, China; ^2^ Liaoning Agricultural Informatization Engineering Technology Research Center, Shenyang Agricultural University, Shenyang, China

**Keywords:** amodal segmentation, image segmentation, transformer, occlusion recover, ecological monitoring

## Abstract

Common object detection and image segmentation methods are unable to accurately estimate the shape of the occluded fruit. Monitoring the growth status of shaded crops in a specific environment is challenging, and certain studies related to crop harvesting and pest detection are constrained by the natural shadow conditions. Amodal segmentation can focus on the occluded part of the fruit and complete the overall shape of the fruit. We proposed a Transformer-based amodal segmentation algorithm to infer the amodal shape of occluded tomatoes. Considering the high cost of amodal annotation, we only needed modal dataset to train the model. The dataset was taken from two greenhouses on the farm and contains rich occlusion information. We introduced boundary estimation in the hourglass structured network to provide *a priori* information about the completion of the amodal shapes, and reconstructed the occluded objects using a GAN network (with discriminator) and GAN loss. The model in this study showed accuracy, with average pairwise accuracy of 96.07%, mean intersection-over-union (mIoU) of 94.13% and invisible mIoU of 57.79%. We also examined the quality of pseudo-amodal annotations generated by our proposed model using Mask R-CNN. Its average precision (AP) and average precision with intersection over union (IoU) 0.5 (AP50) reached 63.91%,86.91% respectively. This method accurately and rationally achieves the shape of occluded tomatoes, saving the cost of manual annotation, and is able to deal with the boundary information of occlusion while decoupling the relationship of occluded objects from each other. Future work considers how to complete the amodal segmentation task without overly relying on the occlusion order and the quality of the modal mask, thus promising applications to provide technical support for the advancement of ecological monitoring techniques and ecological cultivation.

## 1 Introduction

Currently, the global increase in food demand, coupled with a shortage of labor and insufficient food supply, has posed significant challenges (Tian et al., 2021). The use of automation-based growth detection systems and intelligent harvesters has emerged as a primary solution in modern agriculture. For production control and biomass detection, the morphological information of plant fruits is indispensable. Monitoring the yield and size of crops is crucial for refining seeding and fertilization strategies. Information about the appearance of fruits can aid mechanical equipment in precisely determining the position and shape of the fruits. Traditional machine vision systems encounter difficulties in accurately estimating the dimensions of obscured objects during the image collection process. Similarly, laboratory equipment based on three-dimensional image reconstruction (Wang et al., 2017) also poses challenges when applied to agricultural production activities. Tomatoes play a vital role as a global economic crop. Numerous countries engage in the cultivation and export of tomatoes and their various products, such as sauce, canned goods, juice, and dried tomatoes. This has profound implications for agricultural economies and trade, establishing tomatoes as a key agricultural product across fields worldwide. With the emergence of deep learning, computer vision-based crop detection systems have gained extensive application in various agricultural tasks, including tomato harvesting (Guan et al., 2022), disease identification (Dhaka et al., 2021), and growth monitoring. Kanna et al. (2023) utilized CNN models to train and predict on a dataset consisting of four types of cauliflower diseases, achieving the highest accuracy in validation tests across multiple transfer models. Their work emphasizes the crucial role of advanced CNN models in automating the detection and classification of cauliflower diseases. Kundu et al. (2021) proposed a framework that combines Internet of Things (IoT) with deep transfer learning for detecting and classifying rust and blight diseases in pearl millet, demonstrating excellent accuracy. However, most methods used in detection systems are primarily suitable for tasks such as crop counting (Dolata et al., 2021), leaf counting (Wang et al., 2023), providing limited information about the fruits.

Recently, we have come across several deep learning models focused on tomato detection and recognition. Wang et al. (2022) applied MatDet, introducing a path aggregation network to address issues related to inaccurate bounding box localization and tomato ripeness detection. Patil and Manohar (2022) utilized an enhanced radial basis function neural network (ERBFNN) to improve the efficiency and accuracy of tomato crop leaf disease segmentation. Shinoda et al. (2023) used the Transformer and CNN models as the backbone of Mask R-CNN (He et al., 2018) to determine the location and ripeness of tomatoes with high accuracy. However, for the models mentioned above, both learning and prediction rely on visible objects in the image. These models demonstrate a weakness in predicting the invisible parts and lack the capability to measure the size information of the fruits. The ability to perceive the complete shape of occluded objects is referred to as amodal prediction. Currently, amodal segmentation has become a crucial method for recovering the shape of occluded regions. AmodalMask (Zhu et al., 2017) is a deep neural network that predicts amodal masks using image patches. It serves as a focal point in the research on amodal instance segmentation and represents an extension and improvement of the SharpMask model (Pinheiro et al., 2016). ORCNN (Follmann et al., 2019) is built upon the Mask R-CNN framework, introducing two additional segmentation branches for separately predicting masks of the visible and occluded regions. Subsequently, it calculates the difference between these two masks to generate the prediction for the amodal mask.

Previously, amodal segmentation techniques were primarily applied in the fields of autonomous driving (Qi et al., 2019) and scene understanding (Mohan et al., 2022). In automated agricultural systems, the use of amodal segmentation technology aids in accurately detecting and identifying plants, even when partially occluded by other plants, by recognizing complete plant contours. For detecting and categorizing plant diseases, amodal segmentation technology assists in identifying infected plant portions. Automated agricultural systems require counting fruits and leaves for yield estimation or disease monitoring, and amodal segmentation technology provides an effective solution for this. In the agricultural realm, Chen et al. (2022) leveraged the robust feature extraction and reconstruction capabilities of convolutional autoencoders to recover pixels in obscured regions. To address the challenge of reduced accuracy in detecting obscured citrus fruits, the convolutional autoencoder skillfully extracted meaningful features from the surrounding background information, restoring the integrity of the original image. Gené-Mola et al. (2023) implemented an approach that integrates modal and amodal segmentation based on convolutional neural networks (CNN) in RGB-D images. They proposed a resilient method for estimating fruit size and visibility, specifically tailored for the amodal segmentation of apples. Blok et al. (2021) utilized ORCNN to segment both the visible and amodal regions of broccoli heads, enhancing the size estimation of occluded broccoli heads. Chen et al. (2021) proposed a machine vision approach to locate and grasp ripe tomatoes in complex environments. Kim et al. (2023) introduced a U-Net-based reconstruction network for cucumber segmentation and occlusion recovery. The model exhibited accuracy, with an average precision (AP) of 49.31 and an average precision with intersection over union (IOU) of 0.5 (AP50) at 82.39. Gan et al. (2022) introduced a novel structure based on Attention Graph Convolutional Networks (AGCS) for piglet contour and amodal mask prediction.

The semantic labels for the amodal dataset proposed by Qi et al. (2019) are generated through imagination by multiple annotation experts. These labels lack authentic information about the true shapes of objects and entail significant human labor costs. For amodal labels, PCNet (Zhan et al., 2020) achieved the completion of amodal masks through self-supervised training of a segmentation network. The model takes only the modal masks (visible masks) as input and introduces occluders on these masks. The goal is to restore the previously occluded modal mask. However, during the completion process, the model becomes excessively reliant on the occlusion order of objects in the image. ASBUNet (Nguyen and Todorovic, 2021) replaced the occluders’ masks used in PCNet with occlusion boundaries, thus eliminating the need for occlusion order. Huang et al. (2023) introduced a semi-supervised generative adversarial network (GAN) for amodal segmentation. This is an AIS (Amodal Instance Segmentation) method designed for piglets in farrowing pens, relying exclusively on a modal dataset. The model attained a mean Intersection over Union (mIOU) of 0.823 for the segmentation of occluded piglets.

In this study, our goal is to decompose the target objects in greenhouse, explore the potential occlusion relationships among highly similar objects, reassemble the scene order, and complete the shapes of occluded objects.The primary contributions of our work are as follows: 1) we synthesized a tomato dataset with occlusion using the mean-value cloning technique and provided detailed amodal label annotations for this dataset. 2) we pioneered the combination and application of Swin Transformer and amodal segmentation for plant fruit images. 3)we proposed a self-supervised partial completion network that acquires the ability to fill in the invisible parts of tomatoes with training solely using the modal mask. 4) we utilized a segmentation loss method combined with GAN loss to enhance the quality of predicted amodal masks.

We proposed an amodal segmentation model based on Swin Transformer (Liu et al., 2021) and boundary estimation. Building upon the improved Swin Transformer UNet (Cao et al., 2023), we adopted a partial completion approach and trained a partial completion network, thereby reducing reliance on a large number of artificial amodal annotations for training. Additionally, we incorporated ideas from occlusion boundary estimation in ASBUNet and adversarial generative learning. We redesigned the prediction weights for occluded and visible regions within our model. This enabled us to enhance the accuracy of occlusion boundary prediction and increase the realism of the resulting amodal segmentation masks. Finally, we used the predicted amodal mask results from our proposed model as pseudo-amodal annotations and fed them into Mask R-CNN for training. This process allows us to evaluate the ultimate quality of the generated pseudo-amodal annotations.

## 2 Materials and methods

### 2.1 Data acquisition

**Figure 1:**
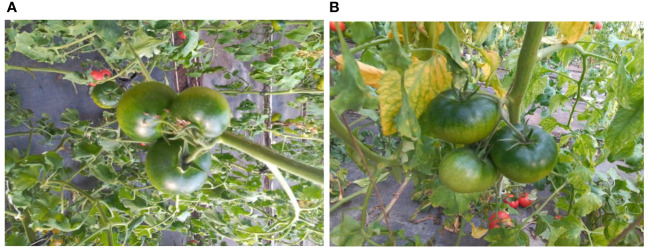
Occlusion scenes in the natural growing environment of tomatoes; **(A)** is obtained from the top view angle; **(B)** is obtained by the slanted side angle.

We selected tomatoes (variety: 325) in the greenhouse as the subjects for our study. To minimize the impact of lighting conditions and shadows, we utilized an additional fill light panel to provide supplementary lighting when necessary. To ensure the dataset includes significant occlusion relationships, we opted for ripe tomatoes as our objects and captured images at various positions and orientations in space. As illustrated in **Figure 1**, to enhance the clarity of occlusion relationships in the image, we intentionally chose scenes with a higher degree of occlusion on the tomato fruits.

We employed an Azure Kinect depth camera to capture tomato RGB image data at a resolution of 1280 x 720 and above. Developed by Microsoft, Azure Kinect is a depth camera device capable of automatically selecting per-pixel gain, facilitating a wide dynamic range that cleanly captures both near and far objects. Azure Kinect primarily relies on structured light technology and utilizes time-of-flight (TOF) techniques to obtain depth information. Additionally, it incorporates other sensors such as an RGB camera and a microphone array to provide a comprehensive perception of data. This technology allows us to acquire both color and depth information of the scene simultaneously in the same space-time.

### 2.2 Amodal annotation and datasets construction

**Figure 2:**
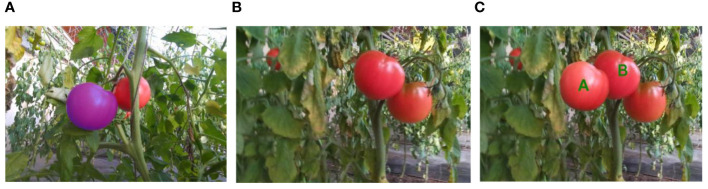
Cloning of source image patches to target image using mean coordinate fusion technique; **(A)** the purple ROIs in the image are the image patches to be cloned; **(B)** target image; **(C)** cloning of the finished image.

Unlike other image segmentation tasks, which typically require pixel-level masking of visible objects, amodal segmentation incorporates scene structures with semantic labels for both visible and invisible parts. The publicly available amodal datasets cocoA and Kinst use ground truth annotated by artificial hypotheses, which would lack the true full shape of the occluded instance. We used a mean-value coordinate fusion algorithm (Farbman et al., 2009) to seamlessly clone the source image patches into the target image, as shown in **Figure 2**. The purple ROI in **Figure 2A**, as the image patch to be cloned, was seamlessly synthesized into the left side of the target image instance B. The image patch was then cloned into the target image. The core idea of this technique is to use the coordinate mapping relationship between the source and target images to interpolate pixel values. An adaptive triangular grid is constructed over the selected image patches, and the interpolated pixel values depend on calculating the mean coordinates of the vertices of each grid, and then sampling the boundary pixels hierarchically so that the number of these vertices is roughly linear to the number of boundary pixels, which translates into solving Poisson’s equation to make a smooth interpolating membrane. This interpolation method maintains the smoothness of the image and is able to handle scale and deformation differences between different images.

We removed all incomplete masks of the masked instances, then combined the patch-synthesized images, and finally removed the images in the dataset that did not have masks, and selected 1000 images. We used the amodal semantic labels of 100 images as a test set, while the modal semantic labels of 900 images were divided into training and validation sets in a ratio of 9:1. To ensure the diversity of the training samples, we used data augmentation techniques such as flipping, panning, and random cropping to triple the number of training samples. After data augmentation, a total of 2,430 augmented training images were accepted.

### 2.3 Amodal segmentation model

The focus of this study is on how to obtain contextual information between occluding objects and decouple the overlapping relationships between them. We aim to consider both the relationship between occluders and occluded objects and estimate the masks for occluders and occluded objects separately.

**Figure 3:**
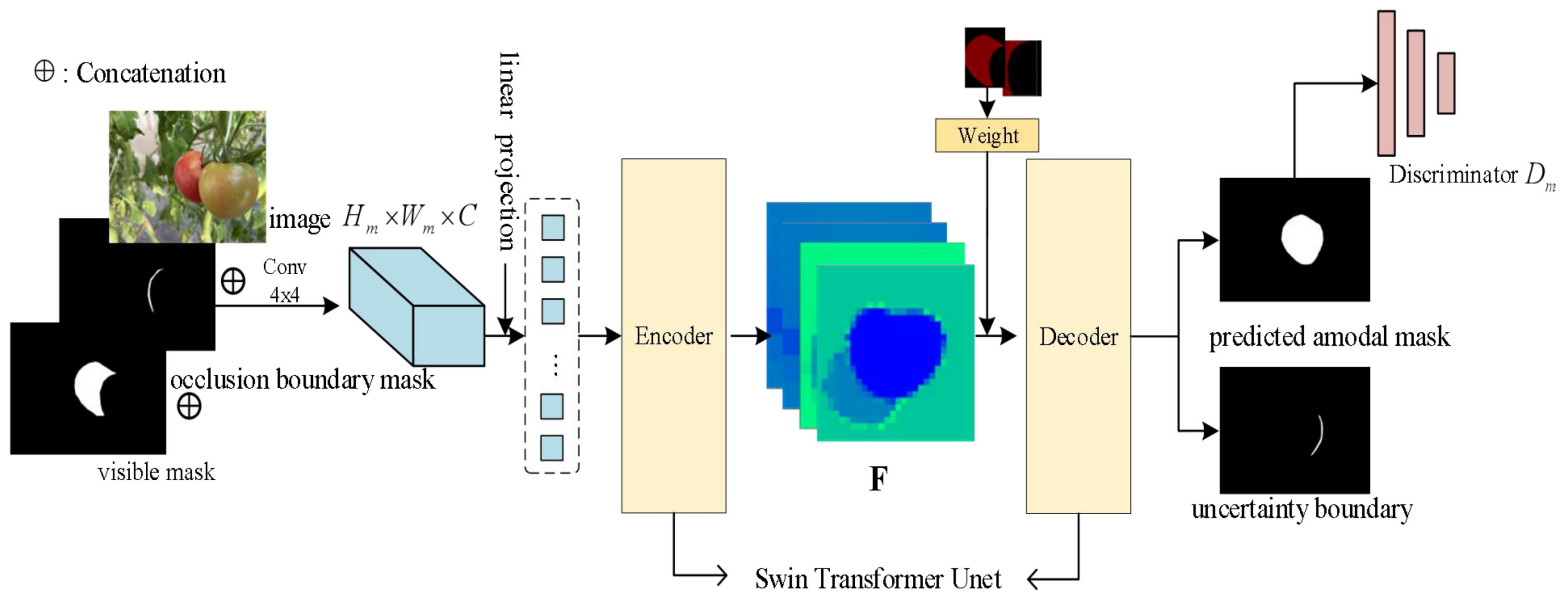
The overview of our approach; F denotes the feature map extracted from the model input by the encoder.

The overview of ACBET (Amodal Completion Network with Boundary Estimation and Swin Transformer Unet) architecture is depicted in **Figure 3**. We used the occlusion boundaries and the primary masks of the visible parts as the inputs of model. It initially processes them with a 4x4 convolutional kernel, resulting in a tensor with 96 channels. Then, we applied a linear layer to transform the dimensionality in the model. After linear mapping, image becomes a series of segmented primary features that are further fed into the encoder. The encoder and decoder in the model utilize a symmetric and improved version of the Swin Transformer Block, based on the Swin Transformer Unet architecture. We modified the Swin Transformer Unet to act as the backbone of the entire network and as the basic segmentation module. The encoder gathers shallow-level features through four sampling layers. These features are then fused with the deep-level features collected by the decoder using skip connections.

In decoding process, we applied a partial completion algorithm to complete the amodal mask. We have improved the partial completion algorithm by introducing joint weights for the occluded region masks and visible region masks. This enhancement strengthens the attention to the interaction between the two parts of information and facilitates the prediction of the entire amodal mask. Finally, through the discriminator learning, the model generates predicted uncertainty boundary maps and improved amodal masks.

#### 2.3.1 Swin Transformer Unet

**Figure 4:**
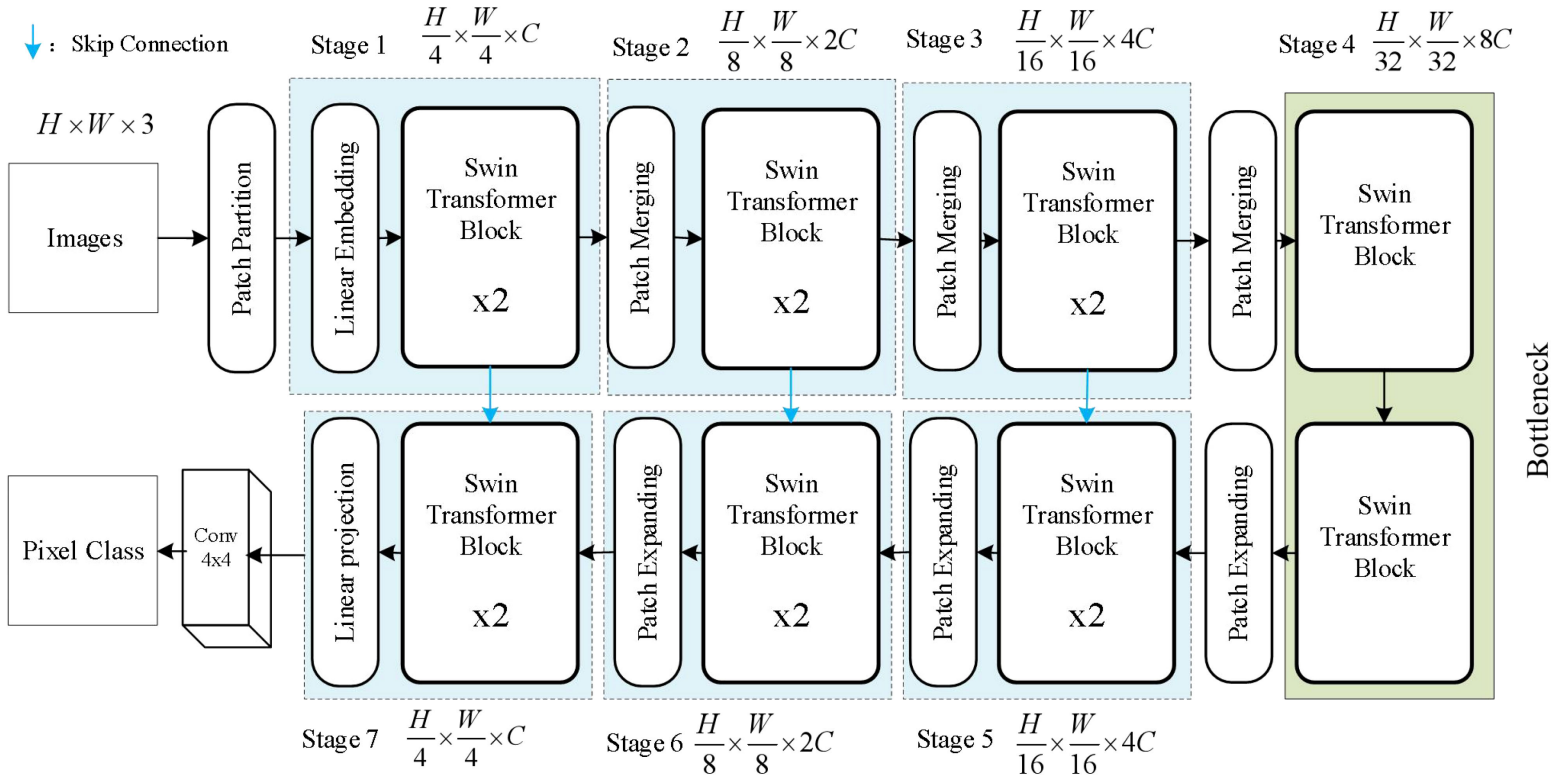
Improved swin transformer Unet structure.

The Swin Transformer architecture is suitable for both general vision backbones and downstream learning tasks. This architecture, by introducing a hierarchical window mechanism, addresses the computational inefficiency issue faced by traditional CNN networks when processing large images. Swin Transformer Unet is primarily composed of Swin Transformer Blocks and designed as a U-shaped symmetric structure (Ronneberger et al., 2015). The architecture can be visualized in **Figure 4**. Swin Transformer Unet first divides the input image into a series of non-overlapping patches, each patch having a size of 4 x 4. Since each pixel has RGB channels, each patch has a dimension of 4 x 4 x 3. Then, each patch is passed through a linear embedding layer to undergo a linear transformation and be transformed into a dimension of C. The input features are then sequentially passed through four stacked modules. In the Patch Merging layer, the network concatenates the patches within a neighboring 2 x 2 range to obtain a feature map with a dimension of 4C. Then, a linear layer is applied to reduce the dimension to 2C. After 4 downsampling modules, the network obtains the feature map F.

When the Swin Transformer Block serves as the decoder, it performs upsampling on the deep features extracting from the previous layer. We add a 1x1 convolutional layer on top of the skip-connected expansion layer to transform the feature size again. Each Patch Expanding layer performs upsampling to restore the feature map to twice its original size.

**Figure 5:**
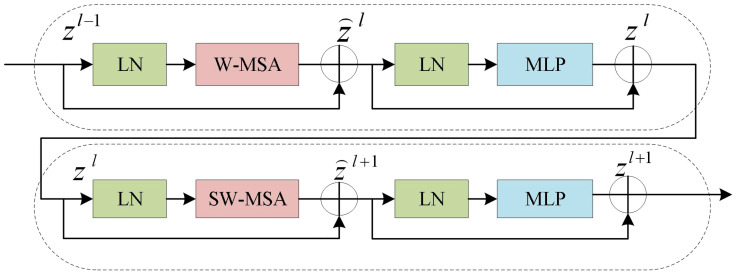
Swin transformer block structure.

However, the final Expanding layer upsamples the feature map to four times the input size. As shown in **Figure 5**, the Swin Transformer Block is composed of two types of structures. Each Swin Transformer Block consists of a relative position encoding layer, a multi-head self-attention layer, an MLP layer, a fully connected feed-forward network layer, and a residual connection.

These blocks can be computed in parallel, improving computational efficiency and significantly enhancing the performance of the model. For each window composed of patches, the W-MSA calculates its Query(Q), Key(K), and Value(V) vectors, following the Self-Attention algorithm. The algorithm can be represented in Equation 1. The Q, K, and V vectors of each window are multiplied by the matrix to obtain the Attention matrix for each window.


(1) $$Attention\left(\text{Q},\text{K},\text{V}\right)=softmax\left(\frac{QK^{T}}{\sqrt{d_{k}}}\right)\text{V}$$

The Attention matrices of all windows are concatenated to obtain the final Attention matrix. To facilitate information interaction between windows, SW-MSA employs a sliding window where each window is shifted to the left or right by a certain distance, allowing for a certain overlap between adjacent windows. Therefore, the information from neighboring windows can be take into account when computing self-attention in the model.

#### 2.3.2 Partial completion algorithm

**Figure 6:**
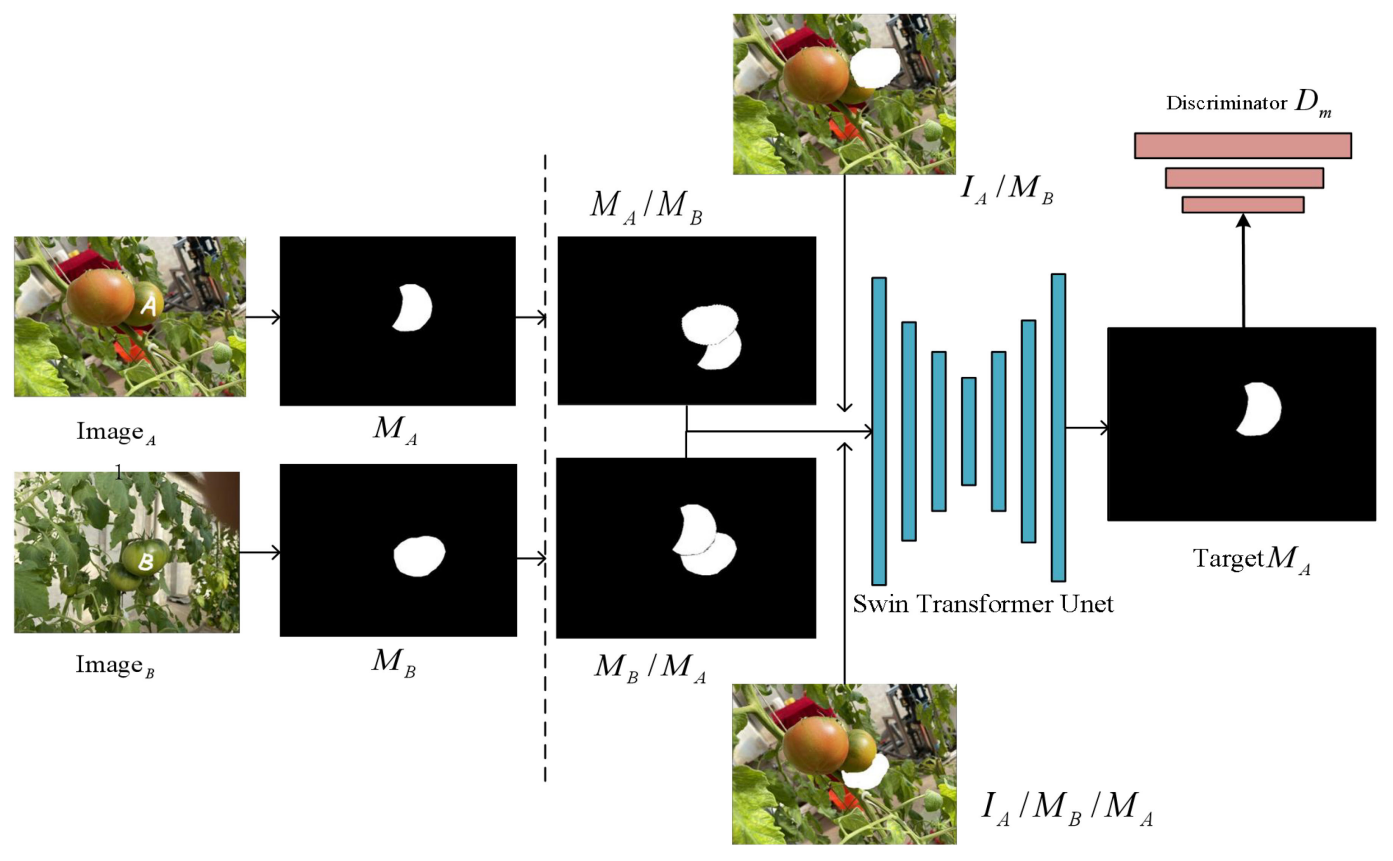
Partial completion of the algorithm’s process. The visible mask of instance A is represented as ${\text{M}}_{\text{A}}$ , and the visible mask of instance B is represented as ${\text{M}}_{\text{B}}$ . When object A occludes object B, it’s denoted as ${\text{M}}_{\text{A}}/{\text{M}}_{\text{B}}$ , and when object B occludes object A, it’s denoted as ${\text{M}}_{\text{B}}/{\text{M}}_{\text{A}}$ .

In previous supervised methods, it was common to incorporate an amodal prediction branch to handle the task of predicting the occluded masks, while another branch focused solely on the visible region’s mask features. Indeed, the completion of the amodal mask requires similar feature information from both the occluded and visible regions. In this study, the training strategies used are partial completion, which is based on the idea of allowing the model to autonomously recover the occluded mask. During the training phase, the images undergo preprocessing, and random sampling is performed to extract the instances of occluders and occluded instances. As shown in **Figure 6**, we randomly selected two images that have an occlusion relationship, denoting the image containing instance A as ${\text{Image}}_{\text{A}}$ and the other image as ${\text{Image}}_{\text{B}}$ . Using existing instance segmentation models or manual annotation, we obtained the visible mask ${\text{M}}_{\text{A}}$ for instance A and the visible mask ${\text{M}}_{\text{B}}$ for another instance B. The partial completion algorithm randomly places the visible mask of one instance on top of the visible mask of the other instance, which can lead to two possible scenarios. The first scenario is that instance A is occluded by instance B. We denoted the mask of the occlusion as ${\text{M}}_{\text{A}}/{\text{M}}_{\text{B}}$ , and the RGB image of instance A occluded by the mask as ${\text{I}}_{\text{A}}/{\text{M}}_{\text{B}}$ . They are combined as input and fed into the network with a hourglass architecture to perform segmentation.

The training objective is to complete the visible mask ${\text{M}}_{\text{A}}$ of instance A. To prevent the model from excessively completing pixels of mask ${\text{M}}_{\text{A}}$ , the second case is that instance B is occluded by instance A, denoted as ${\text{M}}_{\text{B}}/{\text{M}}_{\text{A}}$ . The model combines the mask ${\text{M}}_{\text{B}}/{\text{M}}_{\text{A}}$ and ${\text{I}}_{\text{A}}/{\text{M}}_{\text{B}}/{\text{M}}_{\text{A}}$ as inputs and the objective remains the same, which is to complete the visible mask ${\text{M}}_{\text{A}}$ of instance A. Thus, the model learns to determine whether completion should be performed, achieving the goal of regularization in learning. Subsequently, we introduced a discriminator to improve the quality of the recovered masks. The whole process can be described as follows (Equations 2–4):


(2) $$M_{A},M_{B}=\text{N}\left(\text{A},\text{B}\right)$$

(3) $$M_{preA}=D_{m}\left(PCM_{\theta }\left(M_{A}/M_{B},I_{A}/M_{B}\right)\right)$$

(4) $$M_{preA}=D_{m}\left(PCM_{\theta }\left(M_{B}/M_{A},I_{A}/M_{B}/M_{A}\right)\right)$$

In the above equations, N represents the segmentation model or manual annotation, PCM refers to the partial completion module, and θ represents the parameters of the partial completion module.

#### 2.3.3 Boundary uncertainty estimation

In instance segmentation, applying uncertainty estimation allows quantifying the model’s uncertainty in recognizing instance boundaries in an image. The principle is to introduce a probabilistic model to handle fuzzy and uncertain boundary positions. In the aforementioned partial completion task, when the model recovers occluded instances, the content of the occluded regions may have different pixel fillings due to the uncertainty of the boundaries. As shown in **Figure 3**, the model takes the original image, the mask of the occluded object, and the occlusion boundaries as inputs to the hourglass network module. It then provides a mask branch to handle the occlusion boundaries. The boundary uncertainty map output by the model serves as another boundary for the occluded object, and if the uncertainty is estimated to be high, the uncertainty map with the original occlusion boundary can help the model to capture reasonable shapes and sizes. The modal mask, with a size of 224 x 224 x 2, serves as input to the Swin Transformer Unet. It generates a feature map of size H x W x 2, where H represents the height and W represents the width. The feature map has two dimensions: one channel is used for amodal segmentation prediction, and the other channel is used for estimating the uncertainty of the boundaries. The model calculates confidence intervals to determine the range of predictions. The uncertainty map generated by the model results in lower segmentation loss in regions with higher uncertainty. Additionally, the shape priors obtained from the uncertainty map help the model better understand spatial distribution information. This uncertainty-based boundary estimation optimizes the amodal mask completion task and significantly improves the model’s performance.

#### 2.3.4 Infer paired occlusion order for instances

Humans can intuitively perceive the sequential order of overlapping objects in natural scenes. If we only consider the occlusion of regions of interest (ROI) without incorporating sequential reasoning, it would be insufficient to handle complex scenes with a large number of overlapping objects. Zhu et al. (2017) and Ehsani et al. (2018) considered the depth ordering of instances in the image for the recovery of amodal masks. However, when it comes to handle scenes with cyclic occlusions by ordering the objects, the performance is not satisfactory.

However, when it comes to handle scenes with cyclic occlusions by ordering the objects, the performance is not satisfactory. Zhan et al. proposed a pairwise occlusion relationship sorting approach, where adjacent instance pairs consisting of two connected visible masks represent the occlusion between adjacent objects. However, this method failed to address situations where two objects occlude each other.

**Figure 7:**
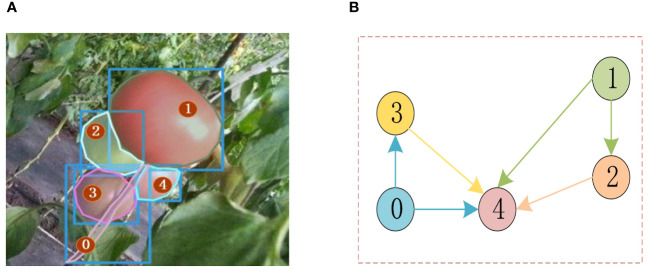
Occluded scene and corresponding sorting graph; **(A)** occluded scene **(B)** corresponding sorting graph.

We drew inspiration from the occlusion boundary estimation method ASBUNet and combined it with the partial completion network to fill in the pixel values of different objects belonging to the boundaries. The combination of these two techniques reduces the errors caused by the partial completion network’s inability to follow consistent object geometry and missing boundaries. We show the relationship between instances using a directed graph of paired occlusion order in **Figure 7**.

G=(Y,T) is used to represent directed graphs with occlusion order. Y denotes the set of all instances in the image with a total of N instances. T is an N x N matrix, where ${\text{T}}_{\text{i},\text{j}}$ represents the occlusion relationship between the adjacent pair of instances ${\text{T}}_{\text{i}}$ and ${\text{T}}_{\text{j}}$ . The calculation formula is as follows Equation 5.


(5) $$T_{i,j}=\left\{ \begin{array}{ll} 0, & if|M_{i}^{A}-M_{i}|=|M_{j}^{A}-M_{j}|=0\\ 1, & if|M_{i}^{A}-M_{i}|<|M_{j}^{A}-M_{j}|\\ -1, & if|M_{i}^{A}-M_{i}|\geq |M_{j}^{A}-M_{j}| \end{array}\right.$$

Where ${\text{M}}_{i}^{A}$ and ${\text{ M}}_{j}^{A}\;$ represent the completed amodal masks for instances i and j. ${\text{M}}_{\text{i }}$ and ${\text{M}}_{\text{j}}\;$ represent the visible masks, and $\left|{\text{ M}}_{i}^{A}-{\text{M}}_{\text{i}}\right|$ and $\left|{\text{ M}}_{j}^{A}-{\text{M}}_{\text{j}}\right|$ represent the pixel value increments for the completed amodal masks. If the increments generated by the mask completion network for instances i and j are equal to 0, it indicates that there is no occlusion between the two instances and they belong to the same layer. In this case, the ${\text{T}}_{\text{i},\text{j}}$ in the matrix would be 0. If the increment of the former is smaller than the latter, it means that I occludes J, and the value of ${\text{T}}_{\text{i},\text{j}}$ is 1. If the increment of the former is not smaller than the latter, it means that J occludes I, and the value of ${\text{T}}_{\text{i},\text{j}}$ is -1. Considering the pairwise occlusion order, we can gradually infer the object order in the entire scene, providing clear prior information and interpretability for amodal mask completion.

#### 2.3.5 Loss function

The output of the model consists of two parts: the predicted amodal mask and the predicted boundary uncertainty map. The result of the amodal mask is obtained by element-wise addition and activation using Sigmoid function (Equation 6), ensuring that the values are within the range of [0,1]. The boundary uncertainty map is obtained by calculating the boundary uncertainty. A higher value of boundary uncertainty indicates greater uncertainty in the model’s predictions of boundary positions, while a lower value indicates greater confidence in the model’s predictions. By generating the boundary uncertainty map, we can quantitatively assess the reliability of the model’s boundary predictions in the image. Therefore, it needs to be non-negative. The result is smoothed and output using Softplus non-linear function (Equation 7).


(6) $$sigmoid\left(\text{x}\right)=\frac{1}{1+{\text{e}}^{-\text{x}}}$$

(7) $$softplus\left(\text{x}\right)=log\left(1+e^{x}\right)$$

Therefore, after introducing the occlusion boundary estimation, we use the loss function:


(8) $$L_{in\_mask}=\frac{1}{\text{N}}\sum_{\text{i}=1}^{\text{N}}\text{γ}\left(m_{i}^{c}=|1\right)L_{i}$$

(9) $$L_{out\_mask}=\frac{1}{\text{N}}\sum_{\text{i}=1}^{\text{N}}\left(m_{i}^{c}=0\right)L_{i}$$

(10) $$L_{i}=\frac{1}{2}\left[{\left(\frac{m_{i}^{t}-m_{i}}{u_{i}}\right)}^{2}+u_{i}^{2}\right]$$

In the above equations, N represents the total number of pixels in the image, and ${\text{m}}_{i}^{c}$ represents the mask of the occluder. In Equation 8, ${\text{L}}_{\text{in}\_\text{mask}}$ represents the loss generated during the mask prediction inside the occluding object, and γ  represents the weight assigned to it. Based on experimental results, we set γ to a constant value of 5. In Equation 9, ${\text{L}}_{\text{out}\_\text{mask}}$ represents the loss for predicting the visible mask outside the occluded region. In Equation 10, the first term of ${\text{L}}_{\text{i}}$ aims to minimize the weighted discrepancy between the predicted amodal mask and the ground truth amodal mask. ${\text{m}}_{i}^{t}$ represents the ground truth amodal mask, while ${\text{m}}_{\text{i}}$ represents the predicted amodal mask. The second term serves as a regularization term for the predicted uncertainty map. Higher values indicate higher levels of uncertainty in the corresponding regions. To improve the quality of amodal mask completion, we also introduced adversarial learning by using a discriminator to minimize the discrepancy between the generated amodal mask and ground truth. The binary cross-entropy loss function was modified as the original method. Its loss function is shown in Equation 11. ${\text{E}}_{{\text{m}}_{\text{i}}}$ and ${\text{E}}_{{\text{m}}_{i}^{C}}$ represent the mathematical expectation in the equation.


(11) $$L_{adv}=E_{m_{i}}\left[log\left(1-D_{m}\left(m_{i}\right)\right)\right]+E_{m_{i}^{c}}\left[logD_{m}\left(m_{i}^{c}\right)\right]$$

The overall loss function L is represented as follows (Equation 12):


(12) $$\text{L}=L_{in\_mask}+L_{out\_mask}+L_{adv}$$

### 2.4 Training and parameters setup

The training consists of three stages. In the first stage, the model is trained with low-resolution images. This stage allows for faster training by using a larger batch size and fewer iterations. In the second stage, the model is trained with high-resolution images in a smaller learning rate. This stage involves a longer training time to ensure better convergence and accuracy on the higher-resolution data. The Swin Transformer has already been pre-trained on ImageNet-1K, and it is recommended to load the pre-trained weights at the beginning of training. Utilizing transfer learning allows the model to more effectively adjust to downstream tasks with limited datasets, particularly when dealing with small fruits. In the third stage, we used Mask R-CNN to test the model’s ability to complement the modal mask. Subsequently, we conducted experiments to evaluate the performance of PCNet, ASBUNet, and our proposed model on the amodal mask completion task. We also compared the accuracy of the respective models on the amodal instance segmentation task. The mask input size of the model is 224 x 224. To accommodate constraints in physical memory and learning adjustments, we conducted 50,000 iterations with an initial learning rate of 5. The batch size was set to 32. For optimization, we employed the SGD optimizer (Stochastic Gradient Descent) with a momentum of 0.9. During backpropagation to optimize the model, a weight decay of 0.0001 was applied. The learning rate schedule includes a learning rate multiplier of 0.1, which adjusts the learning rate for different layers. Every 2000 iterations, predictions are made on the validation set, and the loss is evaluated for validation purposes.

Mask R-CNN is deployed using the open-source framework Detectron2 and PyTorch. We initialized the Mask R-CNN network using the pre-trained weights of the feature extraction network ResNet50 [31]. The parameters of the bounding box regression and fully convolutional networks are randomly initialized. We froze the weights of the feature extraction network and set the learning rate to 0.002 for training the backend network. Training and testing of all models in this study were conducted on one experimental platform to ensure consistency in comparison conditions. The main hardware configuration of the platform includes an Intel(R) Core i9–10980XE CPU with a frequency of 3.0 GHz, 128GB of RAM, and an NVIDIA GeForce RTX 3090 GPU with 24GB of memory. The main software environment includes the Ubuntu 20.04 operating system, PyTorch 1.10 deep learning framework, CUDA 11.7 for general-purpose parallel computing, and cuDNN 8.3.4 for GPU acceleration.

### 2.5 Metrics of evaluation

We used manually annotated amodal masks as the ground truth for verification and testing. For the occlusion order task in inference scenes, we compared the predicted occlusion order diagram with the ground truth occlusion order diagram. We evaluated the accuracy of the entire scene’s pairwise order and the accuracy of pairwise occlusion order using AP-ACC (All Pairwise Accuracy) and OP-ACC (Occlusion Pairwise Accuracy), respectively. In order to evaluate the occlusion order prediction made by the experiments on valid instances, we introduced a strategy where the predicted instances are only evaluated if their IOU (Intersection over Union) with the ground truth mask exceeds a threshold of 0.5. AOP (Average Occlusion Precision) quantifies the accuracy of sequence prediction across various threshold levels. To assess the completion of amodal masks, we compared the predicted amodal masks against the ground truth mask and computed the mean Intersection over Union (mIOU). For pixel classification tasks, we utilized Pixel Accuracy (P-ACC) to evaluate the segmentation model’s quality. For the prediction of invisible mask regions, we also used inv-mIOU as an evaluation metric to measure the accuracy of the predicted masks for occluded regions compared to the ground truth. In the task of amodal segmentation, we used Average Precision (AP) to represent the average precision at different IOU thresholds. AP evaluation is commonly used with IOU thresholds of 0.5 and 0.75, using Average Recall (AR) as an additional evaluation metric. The equation of OP-ACC and IOU are defined as follows (Equations 13, 14):


(13) $$\text{OP}-\text{ACC}=\frac{\sum_{\text{AB}}\left({\text{O}}_{\text{AB}}=1{\text{ and O}}_{\text{AB}}^{\text{pre}}=1\right)}{\sum_{\text{AB}}\left({\text{O}}_{\text{AB}}^{\text{pre}}=1\right)}$$

(14) $$\text{IOU}=\frac{{\text{M}}_{\text{pre}}\cap {\text{M}}_{\text{GT}}}{{\text{M}}_{\text{pre}}\cup {\text{M}}_{\text{GT}}}$$

where ${\text{O}}_{\text{AB}}$ and ${\text{O}}_{\text{AB}}^{\text{pre}}$ denote ground truth and predicted occlusion order.

## 3 Results

### 3.1 Training performance of different models

**Figure 8:**
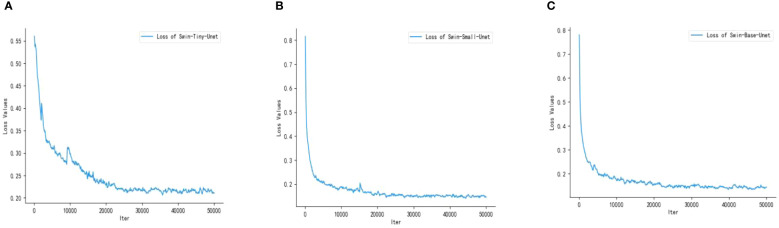
Training loss graphs of the three models; **(A)** Swin-Tiny-Unet **(B)** Swin-Small-Unet **(C)** Swin-Base-Unet.

Swin Transformer includes three network architectures that are designed to adapt to different datasets and tasks based on differences in network depth and the number of attention heads. In this experiment, we have selected three architectures and labeled them with Swin-Tiny-Unet, Swin-Small-Unet, and Swin-Base-Unet. We show the loss profiles of the 3 different architecture models during the training process. As shown in **Figure 8**, the loss curves of the three models exhibit a clear decreasing trend, indicating that all three models have converged during the training process.

Compared to Swin-Tiny-Unet, Swin-Small-Unet and Swin-Base-Unet have a deeper network architecture and a larger number of parameters. However, they converge faster, reaching convergence at approximately 22000 iterations with final loss values of 0.1437 and 0.1448, respectively. Swin-Tiny-Unet converges relatively slower, reaching convergence at approximately 25000 iterations with a final loss of 0.2087. In summary, all three models are capable of completing the learning task. After converging the models to the global optimum using SGD, we selected the best network model based on the performance metrics evaluated on the validation set.

### 3.2 Inferring of occlusion order

Our baseline for the ordering inference task is established based on the Area and Y-axis algorithm (Zhu et al., 2017). The Area algorithm sets a separate heuristic method for the dataset. Since our tomato dataset was captured from various angles and distances, we applied an optimization heuristic that sorts larger instances as foreground instances based on the area of their masks.

The Y-axis algorithm focuses on the bounding boxes of the image instances and sorts them in the order of the detection boxes. Typically, instances with bounding boxes closer to the bottom of the image are placed at the front. In **Table 1**, we presented the results of different methods for the occlusion pairwise sorting task. ACBET achieved higher accuracy in occlusion pairwise sorting compared to PCNet-M and ASBUNet, with an improvement of 5% and 3.3%, respectively.

**Table 1:** Order inference results of different methods (%).

Methods	Input		AP-ACC	OP-ACC	AOP_50_
	mask	image			
Area	√		18.33	39.20	20.40
Y-axis	√		48.33	39.31	40.81
PCNet-M	√	√	91.66	90.19	–
ASBUNet	√	√	93.33	92.15	92.25
ACBET	√	√	96.67	96.07	95.18

The input column is divided into mask input and image input, and the symbol √ represents the corresponding input item.

Compared to the performance of the previous baseline in occlusion pairwise sorting, our model demonstrates higher metrics and performance, with an accuracy of 96.07%. The model achieves an order prediction accuracy of 96.67% for the entire scene. Additionally, at an IOU threshold of 0.5, the sorting accuracy of adjacent instances in the occluded regions reaches a significantly high level of 95.18%, indicating that the model performs better adaptability to the morphology of tomato fruits.

### 3.3 Amodal mask completion

For PCNet and ASBUNet, the occlusion pairwise sorting threshold was set to 0.2. The threshold of amodal mask prediction was also set to 0.2. To ensure a fair comparison, we adjusted the training and testing of both comparative models on this dataset to achieve their best performance. We used the visible masks as the input for ACBET. The evaluation results of amodal mask completion are presented in **Table 2**.

**Table 2:** Amodal mask completion results for different models (%).

Methods	backbone segmentation network	mIOU	P-ACC	inv-mIOU
PCNet	Unet	90.62	91.36	–
ASBUNet	Unet	92.47	94.19	38.99
ACBET	Swin-Small-Unet	92.76	94.06	35.91
ACBET	Swin-Base-Unet	93.56	94.89	43.37
ACBET	Swin-Tiny-Unet	94.13	97.83	57.79

**Figure 9:**
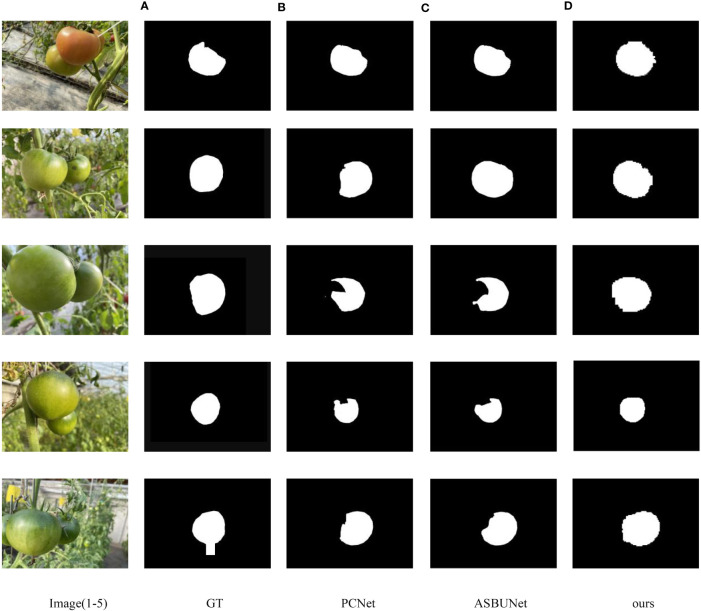
Results of each method for amodal mask completion; The first column from top to bottom, the pictures are recorded as number 1–5 respectively **(A)** GT, **(B)** PCNet, **(C)** ASBUNet, **(D)** ours.

Compared to Swin-Small-Unet and Swin-Base-Unet, Swin-Tiny-Unet shows an improvement in mIOU accuracy of approximately 1.4% and 0.6% respectively. The accuracy of inv-mIOU has improved by approximately 21.8% and 14.4% respectively. Additionally, Swin-Tiny-Unet has significantly fewer parameters both Swin-Small-Unet and Swin-Base-Unet, so it has faster computation and inference speed. In comparison to PCNet and ASBUNet, ACBET with Swin-Tiny-Unet also demonstrates improved mIOU by 3.5% and 1.7% respectively, showcasing better segmentation performance. Moreover, our model achieves an accuracy of 97.83% on the pixel classification task on the test set. Our model outperforms other methods in completing more reasonable amodal shapes when facing similar shapes and colors, as well as moderate to severe occlusions. We visualized the results of the amodal mask completion task in **Figure 9**.

ASBUNet demonstrates reasonable shape recovery for image 1 and image 2. In images 3 to 5, the occluder is placed at the forefront of the image and there is a larger pixel ratio (ratio of pixels between occluder and occluded object), indicating that ASBUNet does not perform well in handling heavy occlusion. While PCNet exhibits varying degrees of pixel missing in all completed amodal masks. Furthermore, our model even predicts amodal shapes that are more interpretable than the manually annotated ground truth. However, the edges around the completed amodal masks are irregular and jagged. That may be caused by pixel stacking resulting from the recombination of pixel blocks after the pixel segmentation by the moving window algorithm.

### 3.4 Results of amodal segmentation

Amodal segmentation task that involves detecting instances from a given image and predicting masks for those instances. The masks represent the complete shape of objects under occlusion perception. We applied the prediction ability of ACBET network in the amodal completion task to our tomato training set, thus We applied the prediction ability of ACBET network in the amodal completion task to our tomato training set, thus obtaining amodal segmentation results and an automatically generated pseudo-amodal annotated dataset.

We trained Mask R-CNN using both the pseudo-amodal annotations generated from ACBET network and the manual amodal annotations. It flanked the quality of the amodal segmentation results and pseudo-amodal annotations generated by our study. **Table 3** presents the comparative results of our experiments. Comparing the mask AP results in the table, our model outperformed PCNet and ASBUNet by nearly 10% and 7.3%, respectively, achieving a score of 63.91%. The AP50 and AP75 were also closer to the results trained with manual annotations, which was significantly better than PCNet and ASBUNet. However, since the dataset consists mostly of close-up images, small objects were not included in the training by default, which also explains the mediocre performance of the APm (AP for medium objects: $32^{2}<\text{ area }<96^{2}$ ) metric.

**Table 3:** Amodal segmentation results of pseudo-amodal annotations generated by different models trained on Mask R-CNN (%).

Training data sources	Box AP	AP50	mask AP	AP50	AP75	APm	APL	AR10
manual amodal	64.92	86.91	66.51	89.12	74.67	42.07	68.94	68.62
PCNet amodal	62.75	88.67	53.52	86.41	59.80	40.24	64.91	58.35
ASBUNet amodal	61.71	88.96	56.65	86.87	66.41	44.68	63.82	61.40
ACBET amodal	60.29	90.01	63.91	86.91	73.52	43.10	64.19	66.18

Box AP and mask AP stand for box average precision and mask average precision, respectively. AP50 stands for average precision when the IOU threshold is 0.5, while AP75 stands for av area <96^2^), and APL represents AP for large-sized objects (area >96^2^). Finally, AR10 refers to average recall with 10 detections per frame.

**Figure 10:**
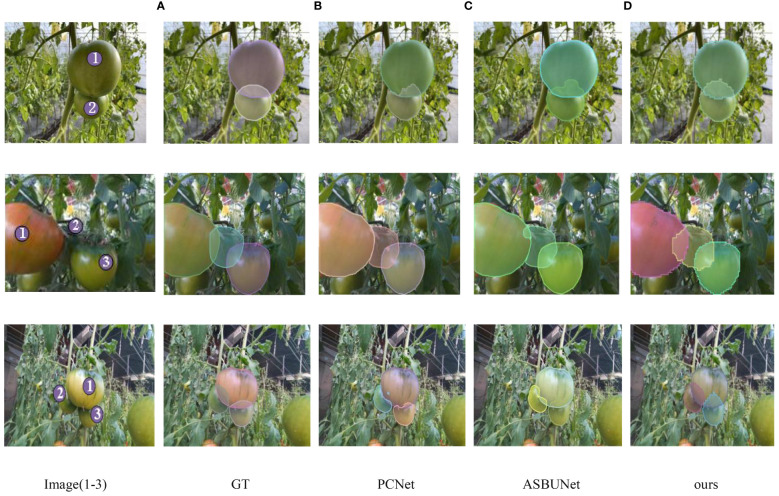
Results of amodal segmentation obtained from PCNet, ASBUNet and ACBET; the first column from top to bottom, the pictures are recorded as number 1–3 respectively; a to d are the results obtained from training and validation of the corresponding models **(A)** GT, **(B)** PCNet, **(C)** ASBUNet, **(D)** ours.

**Figure 10** showed the amodal segmentation results derived from PCNet, ASBUNet and our study. In image 1, all three models are trying their best to complete the full shape of the obscured tomato No. 2, but our model completes a more regular shape. It demonstrates that our model has better performance. In image 2, the tomato labeled as 2 is occluded by the tomato No.1 on the left and tomato No. 3 on the right. Both PCNet and ASBUNet accurately predicted the right side region of the occluded tomato No. 2. When it comes to the No. 2 tomato’s left side region, PCNet chose to ignore it, while ASBUNet had a lower completion rate. Our model is capable of reasonably completing the occluded masks for both sides. In image 3, there are two tomatoes No. 2 and No. 3 occluded by the same object No.1. Our model can simultaneously restore the shape of both targets, while the previous two models tend to complete the mask restoration for only one target and ignore the other.

**Figure 11:**
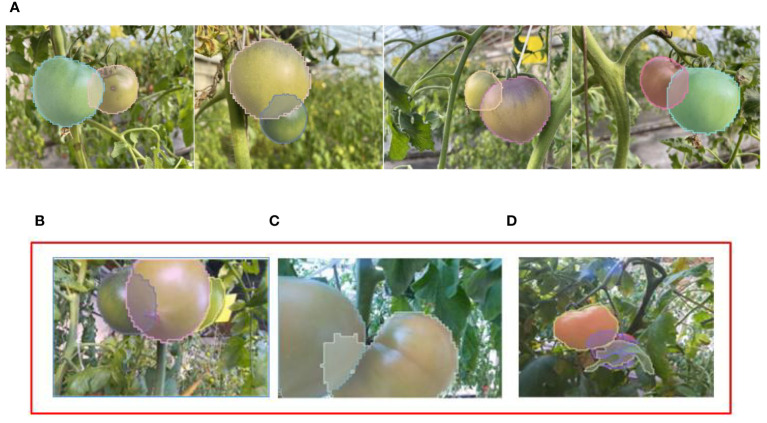
More modal segmentation results obtained by ACBET. **(A)** Results with better amodal segmentation. **(B–D)** Results with poor amodal segmentation.

We further tested the ACBET model on the tomato testing set and obtained additional results, as shown in **Figure 11**. In the first row **Figure 11A**, most of the results demonstrate high-quality predictions of amodal complete shapes. However, there are some results in **Figure 11** that are not as satisfactory.

In **Figure 11B**, we observe that although the occluded tomato instance on the right side is detected accurately, its small visible mask closely resembles that of the leaf. This similarity leads the model to refrain from completing the mask. The occurrence depicted in **Figure 11C** can be attributed to the scarcity of content within the occluded region, causing the model to overly scrutinize pixel completion during training with a substantial dataset. The presence of irregular branches and leaves, coupled with tomatoes from the same class causing occlusion, results in significant obstruction, leading to irregular occluded and visible masks. This complexity compounds the challenge of predicting the scenario depicted in **Figure 11D**.

## 4 Discussion

The occurrence of hidden tomatoes in a greenhouse significantly impacts the precision of tomato detection and counting. Accurate estimation of tomato yield and ongoing monitoring of their growth play a pivotal role in enhancing the economic returns for farmers involved in tomato cultivation. Considering market preferences for fruit freshness, size, and appearance, it becomes imperative to discern the distinct morphology of every tomato during harvesting, even those that are partially obstructed. Mask R-CNN is a standout method in the realm of image instance segmentation, with its primary focus on the visible regions of objects. In a study conducted by Afonso et al. (2020), 123 images were captured using cameras mounted on a rail-guided vehicle. The researchers utilized Mask R-CNN with the RestNext-101 backbone network to segment red and green tomatoes within a greenhouse. The obtained detection metrics reveal an F1-Score of 0.93 for red tomatoes and 0.94 for green tomatoes. Benavides et al. (2020) collected 175 images using a fixed RGB camera. They employed the Sobel operator for tomato edge detection, followed by segmentation based on both color and size (without considering occlusion). The resulting clustered tomato detection rate was 79.7%. Zu et al. (2021) utilized an RGB camera mounted on a mobile robot to capture images, expanding their dataset to 3180 images through the use of data augmentation. They employed the Mask R-CNN with the RestNet-50-FPN backbone network to effectively segment mature green tomatoes, achieving an impressive F1-Score of 0.9284. We integrated the amodal masks predicted by the ACBET model with the images for training the Mask R-CNN featuring ResNet-50-FPN backbone network. This integration aimed to enhance the system’s capability to detect and segment tomatoes of different colors in the images. The outcomes demonstrate that, at an IOU of 0.5, the average segmentation precision on the test set achieved 90.01%. Detailed findings from the mentioned research are presented in **Table 4**.

**Table 4:** Results shown from different studies on tomato fruit detection.

Author	Method	NO. Images	Reported Metrics
Afonso et al., 2020	Mask R-CNN with ResNext-101	123 images without data augmentation	F1-Score of red tomato is 0.93, and green tomato is 0.94
Benavides et al., 2020	Sobel operator for detection, color-based segmentation	175 images	Detection accuracy of beef tomato 90%, and cluster tomato is 79.7%
Zu et al., 2021	Mask R-CNN with ResNet-50-FPN	3180 images without data augmentation	F1-Score of mask for green tomato is 0.92
Ours	ACBET + Mask R-CNN with ResNet-50-FPN	100 images without data augmentation	Detection accuracy of all tomato is 90.0%

The primary contribution of this study lies in restoring the shape of tomatoes under specific occlusion conditions. We introduce an amodal segmentation network based on the Swin Transformer Unet and boundary estimation. By addressing tasks such as occlusion order recovery, amodal mask completion, and amodal segmentation, the model has demonstrated the ability to restore the complete shape of occluded tomato fruits. Currently, datasets containing amodal annotations are scarce, and manual annotation comes with a high cost. For instance, annotating each image in the COCOA dataset (Zhu et al., 2017) takes approximately 18 minutes, while annotating each image in the BSDS dataset (Arbeláez et al., 2011) takes around 15 minutes. To address this issue, we trained a partial completion network to autonomously learn the completion of object pixels without the need for manual amodal annotation during the training process.

In a study by Gené-Mola et al. (2023), amodal segmentation was applied to obscured apples, and diameter estimation and fruit visibility (the ratio of visible pixels to total apple pixels) were based on the predicted amodal masks. The results indicate that, at a confidence level of 0.2, the average precision (AP) for fruit amodal mask prediction is 0.51. Given the model’s inability to directly incorporate depth information from RGBD cameras in greenhouse-captured images, this approach encounters practical limitations. Moreover, the amodal ground truth annotations for the dataset were obtained from 3D tree models generated using Structure from Motion (SfM) and Multi-View Stereo (MVS). Estimation errors might affect the accuracy of the ground truth, rendering it less precise compared to painstaking manual annotations. We provided a test set synthesized through image patching, incorporating the full shapes of tomatoes before they are obscured. This enables us to obtain genuine amodal ground truth, even though the annotations still depend on careful manual labeling. The Swin-Tiny-Unet network utilized in the model incorporates a mobile window-based attention mechanism algorithm, effectively enhancing feature extraction and feature fusion capabilities. The testing results of the ACBET segmentation model on the tomato dataset reveal an average Intersection over Union (mIOU) of 94.13% and a pixel classification accuracy of 97.83%. The average Intersection over Union (mIOU) for invisible mask segmentation reached 57.79%. Introducing uncertainty estimation for occlusion boundaries and incorporating prior information about tomato shapes enhances the accuracy of predicting occlusion order in scenarios with boundary confusion. The average accuracy for predicted pairs of occlusion orders has risen to 96.07%. Additionally, the discriminator in adversarial generative learning, along with its corresponding loss function, contributes to the model generating higher-quality amodal masks.

In **Figures 10** and **11**, our approach presents more convincing prediction images and boundaries when compared to other models. We trained Mask R-CNN on the pseudo-amodal annotated data generated by ACBET, using the validation results on the test set to assess the quality of the pseudo-amodal annotations, and comparing them with manually annotated amodal annotations. Using pseudo-amodal annotations generated by ACBET as the training set, the average precision for mask segmentation reached 63.91%. Evaluation metrics AP50 and AP75 showed a decrease of approximately 2.21% and 1.15%, respectively, compared to the results with manually annotated amodal annotations. This suggests that our model’s predictions for amodal masks closely align with the actual amodal masks. Farming conditions can be intricate, and shadows may be cast on obscured tomatoes due to varying angles of illumination. Furthermore, irregular branches may act as occluders for tomatoes, potentially leading to less distinct boundaries calculated by the model. In our future work, we will concentrate on tackling challenges presented by irregularly shaped occluders. Although our model doesn’t necessitate amodal masks during training, it still depends on modal masks throughout the training process. As emphasized in the work by Zheng et al. (2021), the quality of modal masks significantly influences the completion results of amodal masks. Therefore, improving the quality and accuracy of modal masks will also be a crucial aspect of our future research efforts.

**Figure 12:**
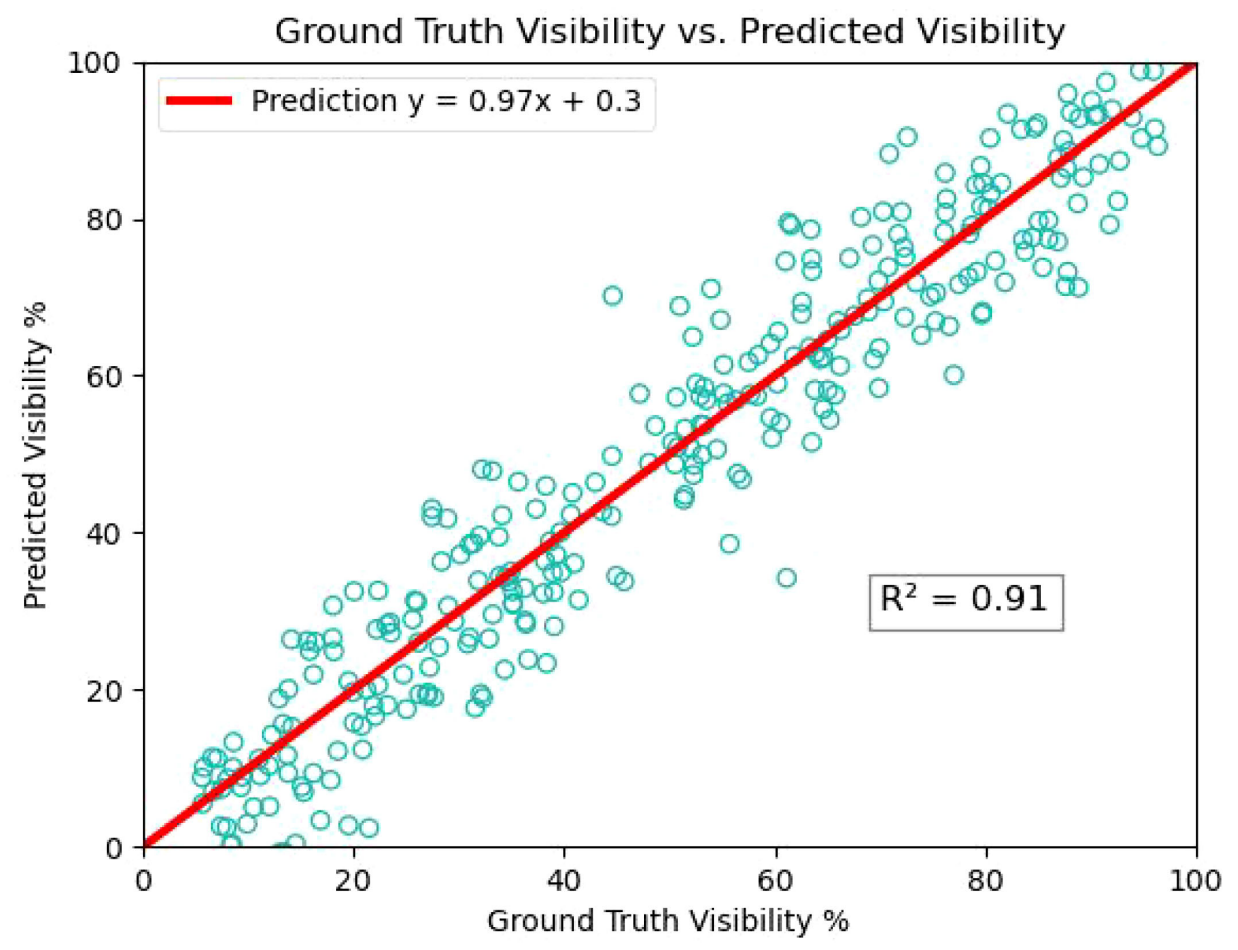
Linear relationship between true and predicted tomato visibility estimate.

Based on the experiments of amodal mask prediction and segmentation in tomato, it can be applied to other fruit and vegetable crops such as apple, maize seedlings and cucumber in the future to further study the plant phenotypes, such as calculating the surface area and volumetric dimensions, which will be beneficial for fruit detection and fruit grading. We estimated the visibility by calculating the ratio of visible pixels (modal mask area) to the total pixels of the tomato (amodal mask area). **Figure 12** shows the experimental results of tomato visibility estimation, with most tomatoes having visibility values in the 15% to 40% and 50% to 90% bins. The amount of data, if large enough, can be an important reference for determining the growing season of tomatoes.

## 5 Conclusion

Our research aims to assist farmers in monitoring the growth stages of tomatoes or provide decision support for robotic fruit harvesting. We have the capability to predict the complete form of obscured objects and estimate tomato sizes even in complex environments. The paper introduces an amodal segmentation network based on Swin Transformer and boundary estimation. Initially, we opted for the Swin Transformer Unet as the segmentation network and subsequently modified the network’s depth and attention mechanisms. This can reduce the model’s complexity. Then, we embedded partial completion network modules and a boundary estimation algorithm into the segmentation network. It aids the model in self-supervised learning to predict and complete amodal masks. Subsequently, we integrated GAN loss into the cross-entropy loss to form a new loss function. We selected undisguised tomatoes from the original dataset and used the mean coordinate cloning algorithm to synthesize obscured tomatoes. Subsequently, we integrated GAN loss into the cross-entropy loss to form a new loss function. We selected undisguised tomatoes from the original dataset and used the mean coordinate cloning algorithm to synthesize obscured tomatoes. This method enabled us to acquire amodal ground truth for tomatoes, as opposed to artificially assumed ground truth. The conclusive experimental results revealed that the model attained an impressive average Intersection over Union (mIOU) of 94.12% for predicted amodal masks, along with a pixel classification accuracy of 97.83%. This study offers valuable insights into advancing fruit harvesting systems and monitoring crop growth status in agriculture. By delving into the complex relationship between plant occlusion and visibility, improvements can be made in automated fruit harvesting technology to enhance efficiency and accuracy. Additionally, the ability to monitor crop growth status aids in timely identification and management of plant health issues, ultimately promoting increased agricultural yield and quality.

## Data availability statement

The original contributions presented in the study are included in the article/supplementary material. Further inquiries can be directed to the corresponding author.

## Author contributions

JY: Conceptualization, Data curation, Methodology, Writing – original draft, Writing – review & editing. HD: Conceptualization, Formal analysis, Methodology, Validation, Writing – review & editing. YFZ: Data curation, Methodology, Supervision, Validation, Writing – review & editing. YCZ: Conceptualization, Formal analysis, Methodology, Supervision, Writing – review & editing. TM: Conceptualization, Investigation, Methodology, Resources, Software, Writing – review & editing.
